# The effect of handedness on spatial and motor representation of pitch patterns in pianists

**DOI:** 10.1371/journal.pone.0195831

**Published:** 2018-05-02

**Authors:** Eline Adrianne Smit, Makiko Sadakata

**Affiliations:** 1 MARCS Institute for Brain, Behaviour and Development, Western Sydney University, Milperra, New South Wales, Australia; 2 Music Department, University of Amsterdam, Amsterdam, The Netherlands; 3 Institute for Logic, Language and Computation, University of Amsterdam, Amsterdam, The Netherlands; 4 Artificial Intelligence Department, Radboud University Nijmegen, Nijmegen, The Netherlands; University of Zurich, SWITZERLAND

## Abstract

This study investigated the effect of handedness on pianists’ abilities to adjust their keyboard performance skills to new spatial and motor mappings. Left- and right-handed pianists practiced simple melodies on a regular MIDI piano keyboard (practice) and were then asked to perform these with modified melodic contours (the same or reversed melodic contour causing a change of fingering) and on a reversed MIDI piano keyboard (test). The difference of performance duration between the practice and the test phase as well as the amount of errors played were used as test measures. Overall, a stronger effect for modified melodic contours than for the reversed keyboard was observed. Furthermore, we observed a trend of left-handed pianists to be quicker and more accurate in playing melodies when reversing their fingering with reversed contours in their left-hand performances. This suggests that handedness may influence pianists’ skill to adjust to new spatial and motor mappings.

## Introduction

Scissors, tin openers and knives …, there are a number of tools specially adjusted for left-handed people. However, the majority of tools in our everyday life are designed for the right-handed population and, in music, it is not common to see an instrument specially designed for left-handed musicians. Left-handed musicians are often using the same instruments as their fellow right handers, while they might benefit from an instrument specifically built for them. Reversed instruments do occur more often in popular music but these are mostly limited to instruments that require different functions for the left and right hand, such as the left-handed guitar. Instruments that require similar functions and movements for both hands, such as keyboards, are rarely designed for left-handers. Interestingly, Jäncke [1] reported such a case of a special instrument, a reversed piano—on which high pitch is on the left and low pitch on the right side of the keyboard. In fact, his study describes a case where a left-handed pianist, C.S., showed a very quick adaptation to such reversed mapping. Notably, Jäncke [1] attributed this adaptation skill to the pianist’s left-handedness. In a follow-up study, it was reported that the level of functional cortical asymmetry could explain this pianist’s outstanding learning skill [2]. Geza Loso is another example of a pianist who fully incorporates the left-handed piano in his musical practices [3]. Given that the left-handed population is always confronted with tools that are designed for right-handed people, it may be possible that they are more flexible in adapting their mappings of internal representations, such as space, motor and pitch height. However, we do not know whether these are representative figures for left-handed pianists. In the current study, we systematically looked into this, namely, the effect of handedness on spatial and motor representation of pitch patterns in pianists.

Theories of the origin of handedness are not portraying the same story. Some say that certain happenings, such as the preferred hand while thumb sucking in prenatal development, play an important role for the development of handedness [4], whereas others attribute the origin to hemispheric specialization [5], or genetic factors [6]. Recent studies started to highlight differences in brain lateralization for left-handed and right-handed populations for various tasks. For example, right hemispheric dominance in language processing increases proportionally with the level of left-handedness (e.g. [7,8]). Such less hemispheric specialization in left-handers has also been confirmed in visual [9] and motor processing domains [10]. Although traditionally handedness has been treated as dichotomy, more recent theories suggest continuity [6] or multi dimensionality [11].

What do we know about the relationship between handedness and musical skills? A number of older studies found higher occurrences of left handers and ambidextrous among musician than non-musician population [12–14]. These studies, however, have not been systematically conducted, as different handedness assessment methods, and different interpretations for the group of musicians were used. For Aggleton, Kentridge & Good [12], musician groups comprised of composers, orchestral instrumentalists and choir members, whereas for Hassler and Birbaumer [14] participants with different levels of musical abilities were used. Because there are no studies having used the same methodology to assess handedness and musicianship, it is difficult to acknowledge results reporting higher levels of left-handedness amongst musicians.

Traditional theories evolved around the idea that music is processed mostly in the right hemisphere [15], creating room for the argument that left-handedness, with right-hemisphere dominance in hand coordination, could therefore be suited to musical activities. Some studies confirm handedness-based asymmetry in music related tasks, e.g. auditory memory tasks [16–18] that handedness may have a systematic effect on musical skills. Kopiez et al. [19] found that for sight reading, motoric laterality with neurobiological advantages was found for non-right-handed pianists.

However, more recent evidence showing that music being processed in multiple areas in both the right as the left hemisphere makes the story not so straightforward. Furthermore, extensive amount of musical practice appears to obscure pure effect of handedness: Kopiez et al. [20] found that musicians’ right hemispheres demonstrate higher temporal precision in general, for both right-handed as well as left-handed musicians. They interpret this that the handedness-based difference is compensated by practice on a non-inverted instrument. The overall story of handedness and its effects on hemispheric specialization for musical abilities is thus inconsistent and incomplete, but described studies here seem to suggest a difference between left- and right-handed musicians at some level.

As far as we know, we do not have much knowledge of whether handedness influences the way musicians dynamically adjust their internal representations of abstract knowledge in performance. Handedness could play a significant role according to studies of Jäncke [1,2] which described the adaptability of a left-handed pianist to the reversed keyboard. The present study tests this hypothesis using a paradigm involving learning and playing of simple melodies on a piano keyboard.

There are two lines of theories explaining how motor movements are related to the learning of a novel melody [21]. Some suggest that the information of an event related to motor movements might be dependent on the instigator of that event [22,23], while another suggests that the information is abstract and therefore a change of motor movements does not affect the event itself [24,25]. Interestingly, previous studies indicated that the encoding strategy differs between novices and advanced musicians. For example, Palmer and Meyer [23] showed that learned motor movement (e.g., contour) plays a more significant role for beginner pianists while abstract concepts (e.g., tonality) are more important for advanced pianists. This indicates that advanced pianists learn melodies through abstract representations rather than motoric memory. Their finding matches with other studies showing that part of the encoding during learning of a pitch pattern sequence happens in an abstract manner independently from the physical motor practice [26,21].

Playing the reversed keyboard requires learning of a new mapping of motor-space-pitch associations. This mapping can be further broken down into two: space-pitch and motor-pitch mappings. It is well-known that we tend to associate pitch height with internal vertical spatiality [27,28]. The association between mental representation of pitch and motor responses has been found in multiple studies [29–33]. For example, responses are quicker when the pitch height of a response target is congruent with response orientation (e.g. high pitched sound with upper key response, low pitched sound with lower key response), which is named as the SMARC effect (Spatial-Musical Association of Response Codes [32]). Interestingly, Rusconi et al. [33] found that musicians show a stronger SMARC effect than non-musicians. This could mean that playing the reversed piano is more challenging for advanced pianists, because they need to perform a mental reverse of well-established space-pitch mapping. Similarly, learning another mapping, the reversed motor-pitch association, can also be challenging, given that advanced pianists are trained extensively in one specific direction of mapping.

Our study investigated whether left-handed pianists are better in learning a new mapping of these extremely well-learned associations than right-handed pianists. In order to compare performance of left- and right-handed pianists, we carried out an experiment based on earlier studies by Palmer and Meyer [21,23]. They investigated pianists’ ability to generalize their learned motor movements using a transfer of learning task. The APA Concise Dictionary of Psychology defines transfer of training as “the influence of prior learning on new learning, either to enhance it or to hamper it” [34]. The direction (positive or negative) and the degree of transfer of learning reflects the ability of abstracting and generalizing learned skills in one situation to another situation [35,36]. It also depends on the similarity between the original and the transfer situation. Often a great transfer of learning is observed when the new skill is very similar to the learned skill (positive transfer), and when the situation does not change (near transfer). Our experimental task asked pianists to transpose a learned melody to different conditions (different hands, different melodic contours, etc.). It was a speeded task, and performance duration of a melody was used as a measure to indicate degree of transfer from practice to test sessions: the shorter the duration, the more fluent the performance. With this setup, we can measure how our two groups of pianists learned new target mappings, namely, new space-pitch mapping and new motor-pitch mapping (see the Method section for details). We hypothesized that reversed mappings (space-pitch and motor-pitch) result in poor transfer and more errors in general, and that left-handed pianists would perform better than right-handed pianists in the reversed conditions based on the previous study by Jäncke [1].

## Methods

### Participants

Twenty-five conservatory piano students of Arnhem (NL) and Antwerp (BE) and the University of Amsterdam (NL) took part in the study (mean age = 23.7 years, sd = 3.8, 13 females). According to self-report, 13 were right-handed and 12 were left-handed. Of the left-handed group 84% had 10 or more years of formal piano training, 6% 6–9 years and 6% 3–5 years. Of the right-handed group, 77% had 10 or more years of formal piano training and 23% 6–9 years. Written informed consent was obtained from all participants, and the study was approved by the Ethical Committee of the Faculty of Humanities of the University of Amsterdam.

### Materials

The Edinburgh Inventory Handedness Questionnaire was conducted, which is an adapted version of the Oldfield handedness questionnaire [37,38]. The questionnaire consists of 15 items in which the participant is asked to put a checkmark for which hand is preferred during activities such as writing and drawing. The outcome number represents the Laterality Quotient [38], which can range from -100 (completely left-handed) to 100 (completely right-handed). The handedness questionnaire results have shown to be highly correlated with those of behavioural tests [39–41], and this index has been widely used. The Goldsmiths Musical Sophistication Index [42] was used to assess participants’ musical background. The questionnaire takes about 10–15 minutes to complete.

**Fig 1:**
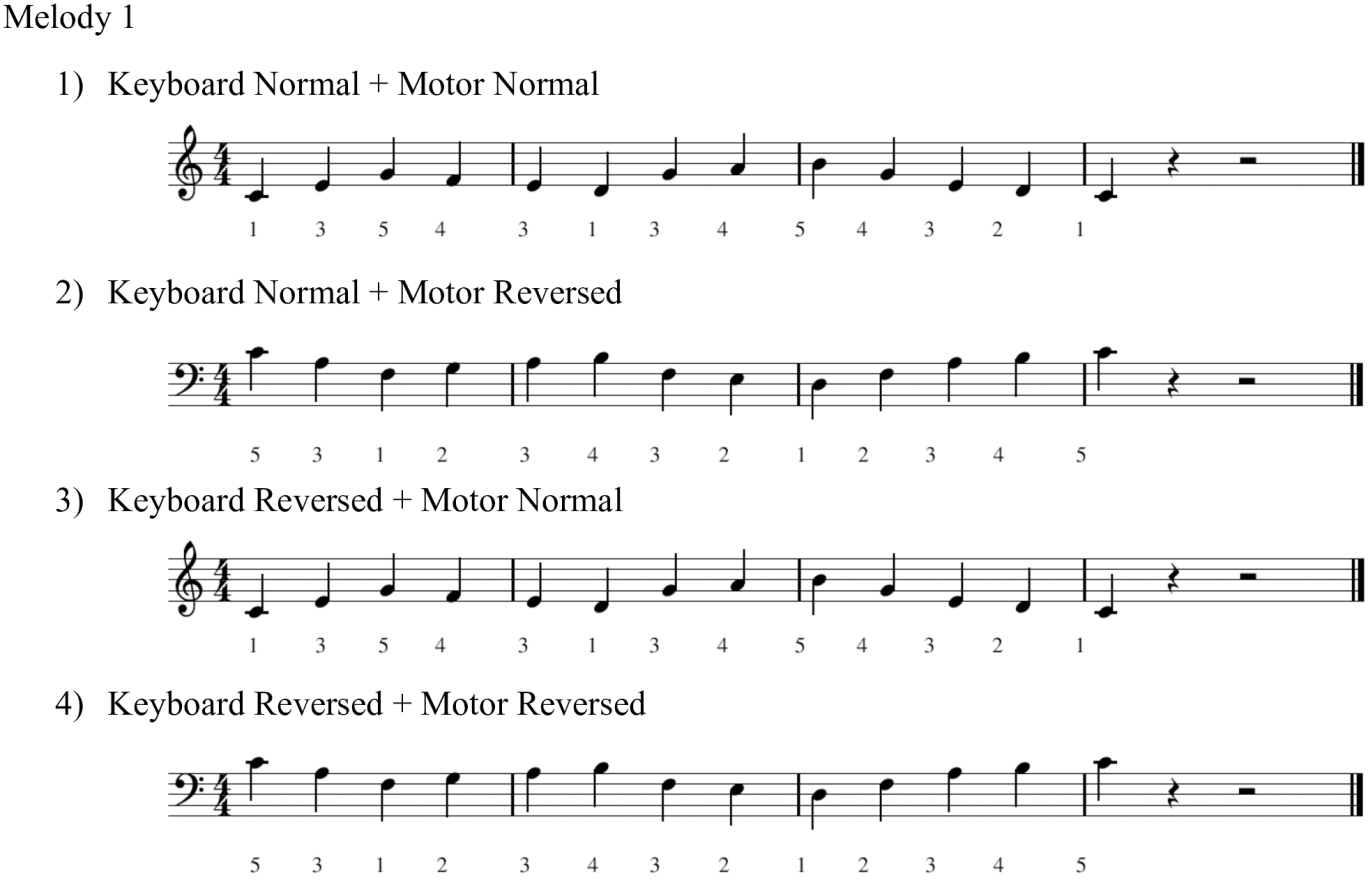
Example of the four conditions of melody 1.

A transfer of learning paradigm, as described in studies of Palmer and Meyer [21,23], was used as the main task. Eight sets of melodies were composed in the same manner as their previous studies (see Fig 1). Melodies consisted of 13 quarter-notes and were all composed in C major with 4/4 meter. Sequences contained one specific difficulty (e.g. one necessary repositioning of the hand), but were overall designed to be relatively simple (i.e. without large intervals, no repetitions of consecutive notes, without black keys). Melodies were designed to have similar level of difficulty.

Skills to adapt to a new space-pitch mapping were tested by comparing two Keyboard conditions (normal / reverse) and to a new motor-pitch mapping were tested by comparing two Motor conditions (normal / reverse melodic contours). One melody was practiced 10 times, and then transposed to the following four conditions in a random order: 1. Keyboard Normal + Motor Normal, 2. Keyboard Normal + Motor Reversed, 3. Keyboard Reversed + Motor Normal and 4. Keyboard Reversed + Motor Reversed. Here, condition 1 is the same as the practiced condition, where the best transfer should be observed. The sound of the melody in conditions 2 and 3 were different from the practice melody, while that in condition 4 was identical to the practiced melody. An example melody with its four conditions for the right hand with corresponding fingering is presented in Fig 1.

### Apparatus

Participants performed the test on a MIDI-keyboard (M-Audio Keystation MIDI 32). A custom-made software recorded onsets and offsets and pitch number of each key press. In order to keep the intervals between the keys similar to the regular keyboard, the reversed keyboard was shifted down with a minor third, e.g. C4 on the regular keyboard would be A4 on the reversed keyboard.

### Procedure

All participants were tested individually. The test was divided in two parts: the questionnaires and the performance test. First, participants filled in the handedness questionnaire and the Goldsmiths Musical Sophistication Index. Second, the participants performed a warming-up exercise and then started the real experiment on the MIDI- keyboard. As a warming up, participants were instructed to play freely on the MIDI-keyboard for 2 minutes. Subsequently, participants played the C-major scale 10 times as quickly as possible on both the normal and the reversed keyboard. The experimental task consisted of two sub-parts, practice of a melody and the transfer tests. During the practice session, participants were presented with one of the test melodies and were instructed to play it 10 times as quickly as possible with the left and the right hand successively. The order of which hand to play first was randomized. During the transfer test phase, the same melody including its variations were presented and they played each of four variations 4 times, again as quickly as possible. All participants were randomly assigned to play two of the eight melodies and the order of transfer conditions was randomized.

The total duration of each melody was recorded (M-duration). M-durations shorter than 500 ms were considered outliers and discarded from the data. Occasionally, participants started a trial but stopped early without finishing. These trials were not included in the analyses and were removed from the data immediately. Mistakes during the course of a trial (omission of notes and mishits) were encoded as errors and their total number was included for the error analysis.

## Results

### Handedness and Goldsmiths MSI

**Fig 2:**
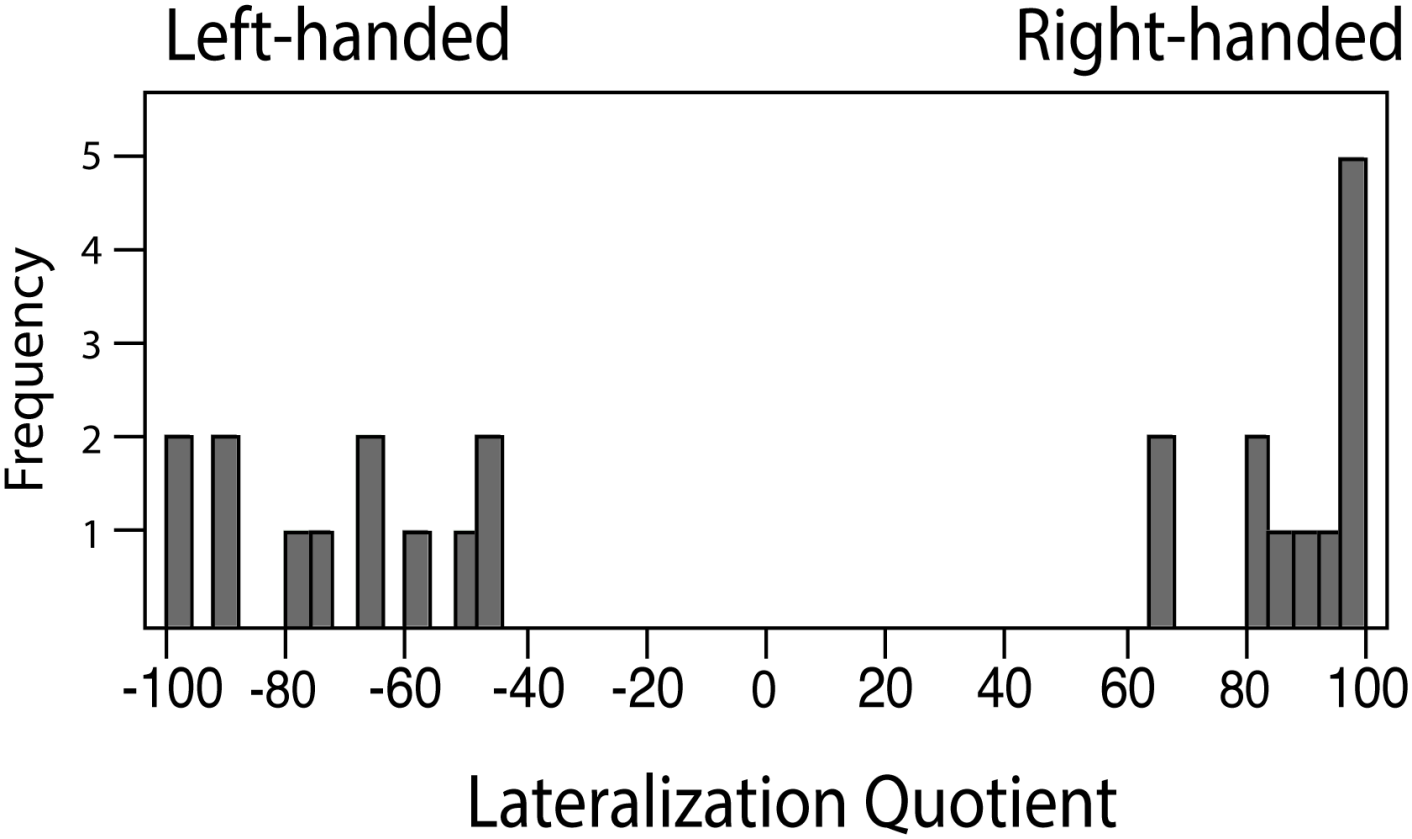
Histogram of Lateralization Quotient scores for left- and right-handed pianists.

The average Lateralization Quotient (LQ) acquired from the handedness questionnaire showed -72.1 for the left-handed and 82.3 for the right-handed group. Fig 2 shows the frequency distribution LQ. One right-handed participant was excluded from the analyses due to a neutral handedness score (LQ = 10). The General Musical Sophistication index was 79.08 for left-handed and 83.67 for right-handed participants and these were not significantly different (t(22) = -1.24, n.s.).

### Transfer of learning task

#### Analysis of M-duration

**Fig 3:**
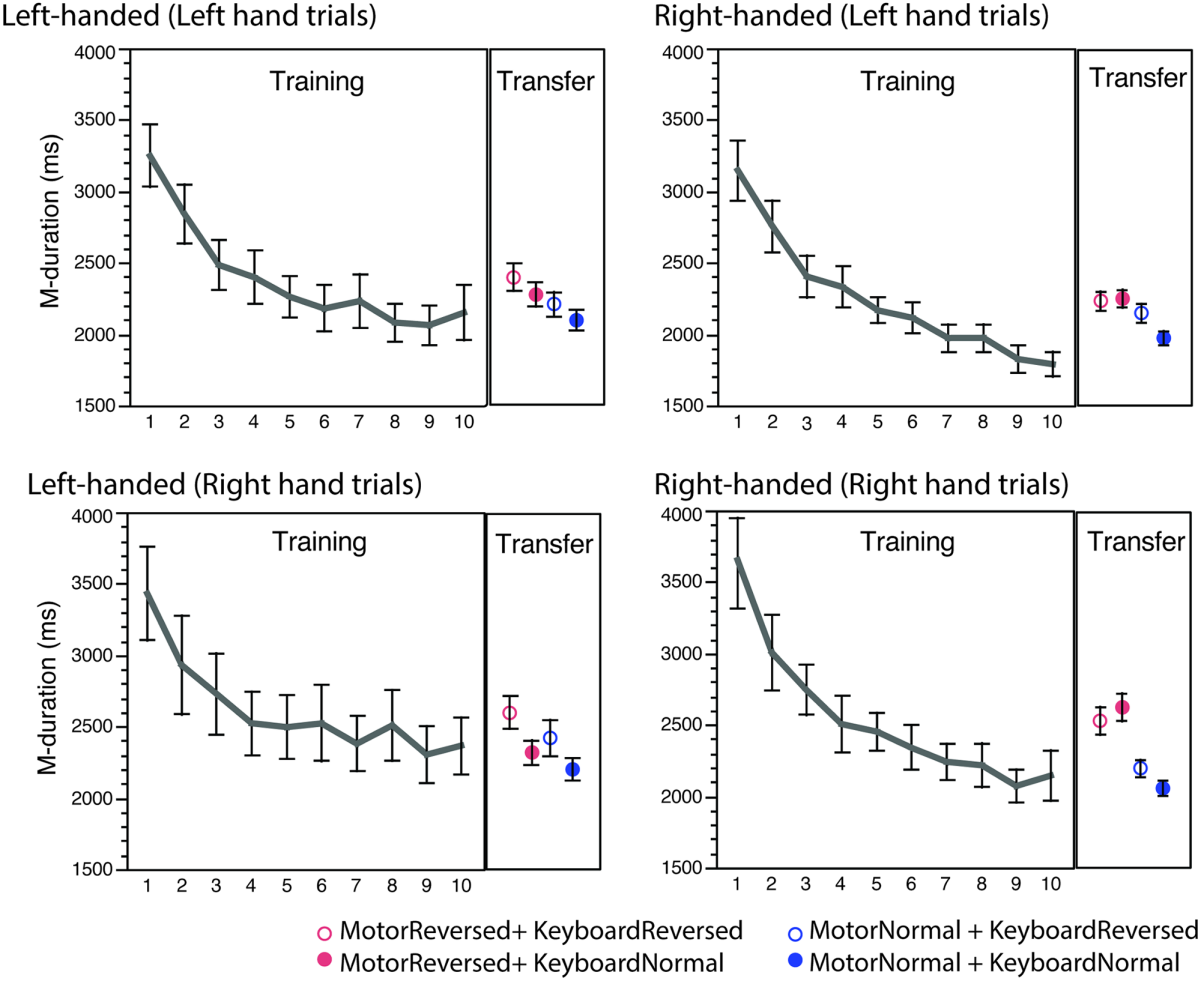
Mean melody duration (M-duration) for 10 practice trials and for 4 transfer conditions. Error bars indicate standard errors.

Fig 3 shows the mean performed melody durations (M-duration) of the 10 practice trials and the averaged M-durations of four transfer conditions. It shows that participants decreased M-durations considerably during the 10 repetitions during practice trials, indicating that they were improving their motor movements (speeding up) over the course of practice. The greater difference of M-durations between the 10^th^ trial of the practice and transfer conditions (average M-duration of four trials each) reflects the degree of transfer.

#### Analysis of transfer performance

**Fig 4:**
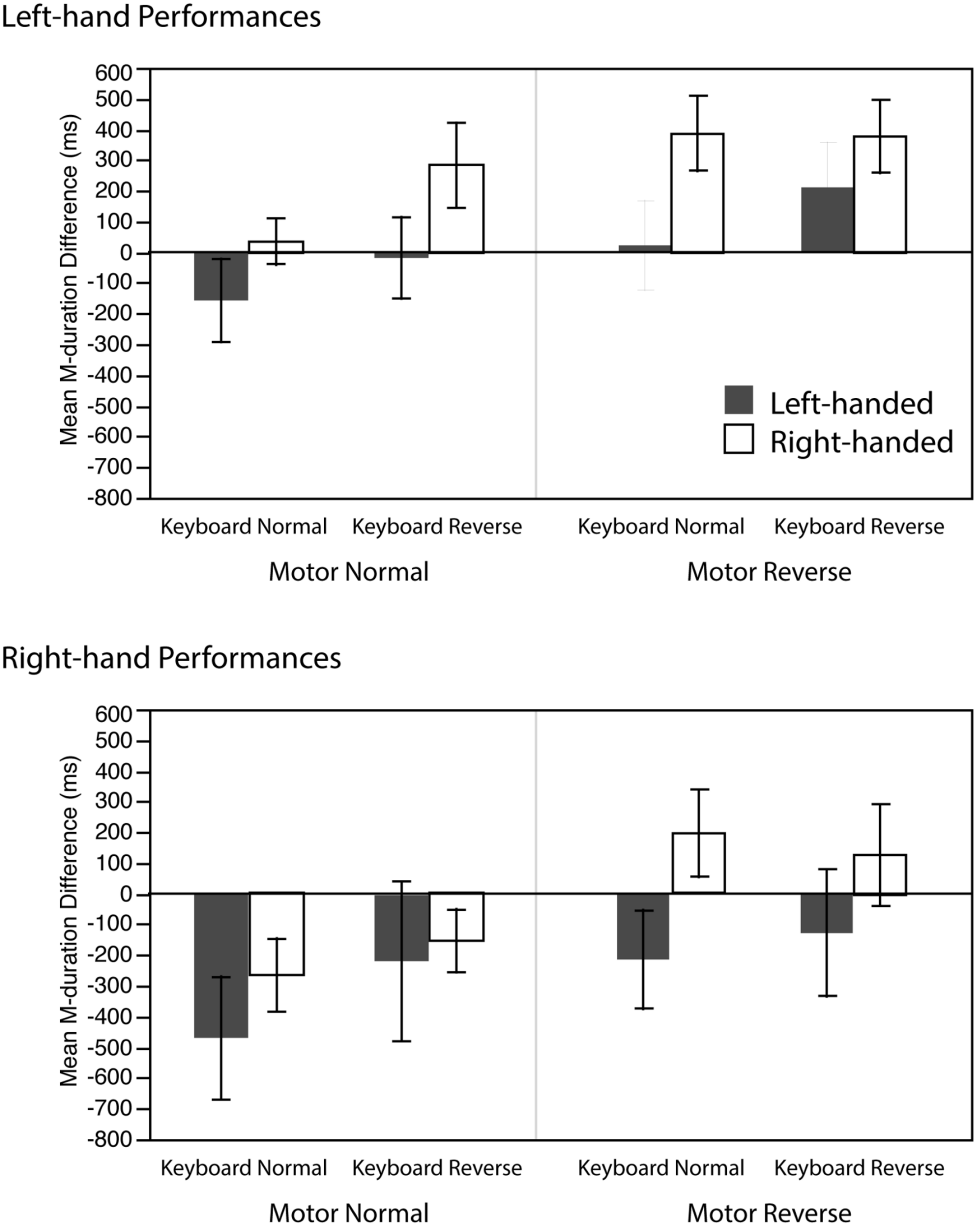
Average M-duration difference of the 4 transfer conditions for left- and right-hand performances. The more negative M-duration difference reflects a stronger transfer of learning effect. Error bars indicate standard errors.

The transfer of learning performance was analyzed by a 3-way mixed ANOVA with *M-duration difference* (the difference of M-durations between the 10^th^ practice trial and average of each transfer condition) as dependent variable and with Handedness (left-/right-handed) as a between-subject factor and Motor condition (normal/reverse), Keyboard condition (normal/reverse) as within-subject factors. This was performed separately for left- and right-hand performances. Fig 4 shows the average M-duration difference for left- and right-hand performances.

The analysis of left-hand performances indicated significant main effects of Motor (F(1,22) = 8.9, p <.01, η_p_^2^ = .29) and Keyboard (F(1,22) = 4.4, p <.05, η_p_^2^ = .17) as well as a marginally significant trend of Handedness (F(1,22) = 3.4, p = .08, η_p_^2^ = .24) without significant interactions among these factors. These suggest that the transfer performance was better when Motor condition was normal (average M-duration difference was 37.1 ms) than when reversed (249.9 ms), as well as when Keyboard was normal (72.4 ms) than when reversed (214.7 ms), and there was a trend of the left-handed group (14.3 ms) outperforming right-handed group (272.8 ms) in performing this task.

The same analysis of right-hand performances again indicated a strong significant main effect of Motor (F(1,22) = 11.0, p <.01, η_p_^2^ = .33) but no significant effects of Keyboard (F(1,22) = 1.3, n.s.) nor Handedness (F(1,22) = 1.2, n.s.). There were no significant interactions. The results indicate that the transfer performance was better when Motor condition was normal (-277 ms) than when reversed (-5.1 ms) but there was no difference with regards to Keyboard conditions and Handedness when performing the task with the right-hand.

A more detailed effect of the degree of laterality on the transfer of learning performance was tested in the form of correlation between the scores of the M-duration difference and the LQ (Lateralization Quotient). However, both right- and left-hand performances did not indicate significant correlations (left-hand performance: spearman’s ρ = .26, n.s., right-hand performance: spearman’s ρ = -.08, n.s.).

#### Error analysis

**Fig 5:**
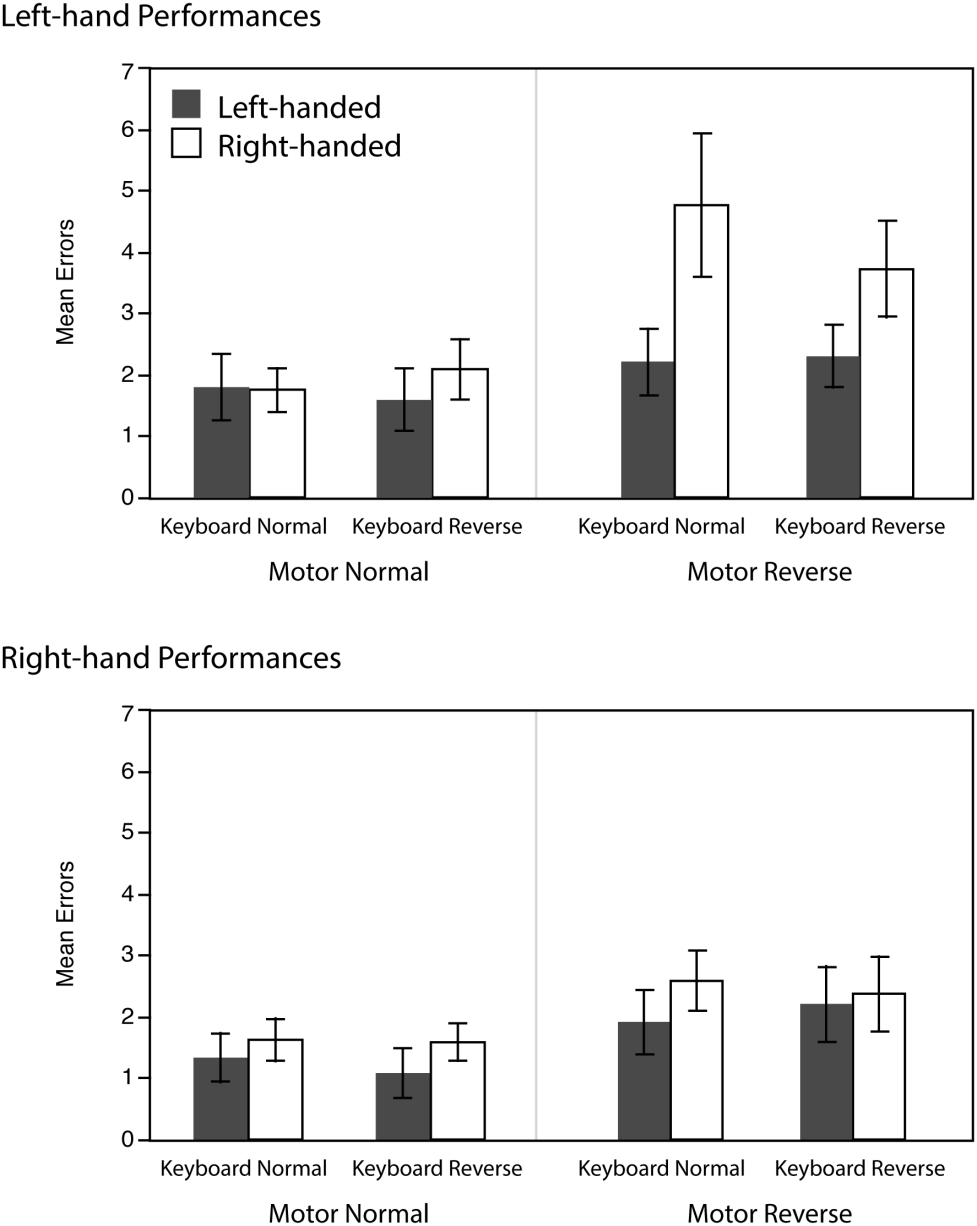
Average number of errors for left-handed and right-handed participants. Two motor conditions are compared (reversed and normal melodic contours). Error bars indicate standard errors.

The error rate was analyzed by the same 3-way repeated measure ANOVA as above, but with the number of errors as dependent variable and with Handedness (left-/right-handed) as a between-subject factor and Motor condition (normal/reverse), Keyboard condition (normal/reverse) as within-subject factors. Fig 5 shows the average number of errors during the test conditions.

The analysis of left-hand error rates indicated significant main effects of Motor (F(1,22) = 18.4, p <.01, η_p_^2^ = .46) as well as a marginally significant trend of Handedness (F(1,22) = 3.2, p = .09, η_p_^2^ = .13) with a significant interactions between them (F(1,22) = 6.8, p <.05, η_p_^2^ = .24). The main effect of Keyboard was not significant (F(1,22) = 0.2, n.s.). No other significant interactions were found. Further simple effect analysis (all Bonferroni corrected) indicated that when Motor condition was reversed, our right-handed pianists made significantly more mistakes than left-handed pianists. Furthermore, right-handed pianists made significantly more mistakes when Motor condition was reversed than when it was normal, while the error rates of left-handed pianists did not differ between two motor conditions.

The same analysis of right-hand performances indicated a significant main effects of Motor (F(1,22) = 7.1, p <.05, η^2^ = .25) but no significant effects of Keyboard (F(1,22) = 0.8, n.s.) nor Handedness (F(1,22) = 0.7, n.s.). There were no significant interactions. The results indicate that the error rate was lower when Motor condition was normal than when reversed and Keyboard conditions and Handedness did not change this trend.

As in the transfer of learning analysis, a more detailed effect of the degree of laterality on the number of errors was tested by computing spearman’s correlation between the LQ score and the number of errors. Here, both right- and left-hand performances did indicate significant correlations (left-hand performance: spearman’s ρ = .37, p = .01, right-hand performance: spearman’s ρ = .29, p = .04). These results suggest that individuals with a stronger tendency of left-handedness performed the task with less mistakes in general.

## Discussion

The current study investigated the effect of handedness on spatial and motor representation of pitch patterns in pianists. Using a transfer of learning paradigm with speeded performance of short melodies on a piano keyboard, we tested how well participants were able to transpose practiced melodies to reversed spatial-pitch mapping and motor-pitch mapping. The results indicated an interesting trend that left-handed pianists showed greater transfer of learning than right-handed pianists especially when the melodies were performed with the left-hand. The results also indicated that transposing melodies to reversed mapping of motor-pitch association (reversed melodic contour) was more challenging than that of spatial-pitch association (reversed keyboard). Furthermore, the error analysis highlighted that the right-handed pianists made significantly more mistakes when they performed the reversed motor condition with their left hand, which apparently was not too hard for the left-handed pianists. Altogether, our results seem to suggest a systematic effect of handedness on the way pianists learn a melody and dynamically apply learned knowledge in performance.

One especially notable finding was that relatively smaller effects of the reversed keyboard were found, as compared to the motor-reverse manipulation. Interestingly, performing on a reversed keyboard was harder for participant’s left-hands, but our finding that pianists performed equally well on normal and reversed keyboard conditions with their right hands seems to contradict previous research, for example, a more robust spatial-pitch mapping in experienced musicians [33]. One possible explanation is that they did not have to deal with new spatial pitch mapping per se: our participants could focus only on practiced motor patterns and ignore sounds produced as a result of key presses. If so, our reversed keyboard condition may not be capturing all aspects that are required to perform the true reversed keyboard described in Jäncke et al. [1,2], where the produced sounds had to correspond with motor commands. Prior work on altered auditory feedback in skilled pianists suggests that the performance of well-learned pieces does not depend on presence or absence of auditory feedback [43–45]. Other studies however suggest that altered auditory feedback interferes with performances and motor actions [46,47]. A follow up study where the focus is on produced sound with the reversed keyboard would be able to address these issues.

Another important discussion point is identification of handedness, which is not a straightforward task. Because of cultural and social pressure (e.g. [48]), there are always some left-handed people who are adjusted to be right-handed. Kopiez et al. [49] proposed a new behavioural measure to distinguish such “right-preferred non-right handed” and “true right-handed” populations. Along with Annett’s right shift theory [6], the proposed measure takes into account the difference in performance regularity and fatigue between right- and left-hand tapping tasks. Also, Büsch et al. [11] emphasized an importance of identifying mixed-handers. Although many studies, including ours, still rely on traditional self-declaration based unidimensional classification of handedness, we think that incorporating such new measures is very important.

The melodies in the current study were composed to have shared music theoretical characteristics, such as duration and structure (no large leaps, repetition of the same note, etc.) in order to maintain the physical difficulty of performance. However, we did not use specific criteria to regulate the musical quality of melodies, although a care was taken to keep it constant. Therefore, there is a possibility that some melodies sounded more pleasant than the others. At least we tried to eliminate such melody specific effect by assigning a subset of them in a random manner and participants did not report certain melodies to be more difficult to play or to reverse than others.

Beginner left-handed pianists have shown to indicate a preference toward the reversed keyboard whereas experienced left-handed pianists prefer a regular keyboard [50]. This agrees with the idea that the regular keyboard may not be optimal for the left-handed population, and that extensive training of bimanual use can correct bias of handedness in motor control [20]. Interestingly, our study was able to catch reminiscence of such handedness differences that persisted through extensive piano training. Our task was applying learned melodic knowledge to different motor commands. Considering that left-handers often have to adapt to right-handed situations, for example by being forced to use right-handed tools, this may not be too surprising: left-handers might adapt learned skills more easily to different motor situations, musical or non-musical.

The effect of bimanual training on motor coordination differences for left- and right-handed population is a promising future topic in music education. So far, left- and right-handed learners of musical instruments have received identical training instructions. Gaining more understanding of systematic differences in these populations will open the door to more effective training methods. Extending our study with less-advanced pianists would be one way to pursue this.

To conclude, the current study presents the potential systematic effect of handedness in pianist’s ability to abstract and adjust the mental representation of a melody. This could suggest that internal representations of a melody are more fluid for left-handed than for right-handed pianists, although we need to interpret the results with care because of somewhat modest effect sizes. The source and the extent of this effect is not fully known and should be further examined. Future studies with musicians, especially ones focusing on mental representation of motor control, should perhaps pay attention to handedness as an important factor.
